# High Child Mortality and Interventions Coverage in the City of Dar es Salaam, Tanzania: Are the Poorest Paying an Urban Penalty?

**DOI:** 10.1007/s11524-023-00813-z

**Published:** 2024-01-12

**Authors:** Sophia Kagoye, Jacqueline Minja, Luiza Ricardo, Josephine Shabani, Shraddha Bajaria, Sia Msuya, Claudia Hanson, Masoud Mahundi, Ibrahim Msuya, Daudi Simba, Habib Ismail, Ties Boerma, Honorati Masanja

**Affiliations:** 1 National Institute for Medical Research, Mwanza, Tanzania; 2 Ifakara Health Institute, Dar Es Salaam, Tanzania; 3 Federal University of Pelotas, Pelotas, Brazil; 4 Kilimanjaro Christian Medical University College, Kilimanjaro, Tanzania; 5 London School of Hygiene and Tropical Medicine, London, UK; 6 University of Dar Es Salaam, Dar Es Salaam, Tanzania; 7 Muhimbili University of Health and Allied Sciences, Dar Es Salaam, Tanzania; 8 Ministry of Health, Dodoma, Tanzania; 9 University of Manitoba, Winnipeg, Canada

**Keywords:** Child Mortality, Urban Penalty

## Abstract

The ‘urban penalty’ in health refers to the loss of a presumed survival advantage due to adverse consequences of urban life. This study investigated the levels and trends in neonatal, post-neonatal and under-5 mortality rate and key determinants of child survival using data from Tanzania Demographic and Health Surveys (TDHS) (2004/05, 2010 and 2015/16), AIDS Indicator Survey (AIS), Malaria Indicator survey (MIS) and health facility data in Tanzania mainland. We compared Dar es Salaam results with other urban and rural areas in Tanzania mainland, and between the poorest and richest wealth tertiles within Dar es Salaam. Under-5 mortality declined by 41% between TDHS 2004/05 and 2015/2016 from 132 to 78 deaths per 1000 live births, with a greater decline in rural areas compared to Dar es Salaam and other urban areas. Neonatal mortality rate was consistently higher in Dar es Salaam during the same period, with the widest gap (> 50%) between Dar es Salaam and rural areas in TDHS 2015/2016. Coverage of maternal, new-born and child health interventions as well as living conditions were generally better in Dar es Salaam than elsewhere. Within the city, neonatal mortality was 63 and 44 per 1000 live births in the poorest 33% and richest 33%, respectively. The poorest had higher rates of stunting, more overcrowding, inadequate sanitation and lower coverage of institutional deliveries and C-section rate, compared to richest tertile. Children in Dar es Salaam do not have improved survival chances compared to rural children, despite better living conditions and higher coverage of essential health interventions. This urban penalty is higher among children of the poorest households which could only partly be explained by the available indicators of coverage of services and living conditions. Further research is urgently needed to understand the reasons for the urban penalty, including quality of care, health behaviours and environmental conditions.

## Introduction

The urban advantage in health is generally attributed to better access to health services, improved infrastructure and greater economic opportunities, as well as lower fertility and fewer high-risk births [1, 2]. In general, these advantages have translated in better child survival for urban children than rural children in sub-Saharan Africa [1]. United Republic of Tanzania, however, is one of the exceptions where urban and rural mortality in children under 5 years is similar, according to national surveys [3, 4].This is partly driven by higher urban than rural neonatal mortality, especially in the first week of life, for which no satisfactory explanation has been found beyond potentially deficits in the quality of maternity care, which are, however, also observed elsewhere in Tanzania [5–7]. Reduced survival benefits from living in the city have also been observed for adults in sub-Saharan Africa, including Tanzania, with higher mortality among adults in urban households than rural households mainly due to the burden of non-communicable diseases (NCDs), co-existence between NCDs and infectious diseases (double burden of disease) and road traffic accidents [8, 9]. This loss of a survival advantage due to adverse consequences of unhealthy physical and social conditions often found in cities has been termed as the ‘urban penalty' [10, 11].

Dar es Salaam is the largest city in Tanzania with an estimated population of 5.4 million people in 2022 census [12], equivalent to 8.7% of Tanzania’s total population. Several decades of rapid urbanization, at an estimated annual growth rate of 5–7%, have led to widespread unplanned growth of the city [13]. In 2013, it was estimated that about 70% of the city’s population lived in informal settlements [14]. Dar es Salaam, however, does not have large contiguous settlements that can be characterized as slums. Rather, the city has a diffuse pattern of informal settlements spread out over an ever-expanding area [15].

The health services in Dar es Salaam are predominantly public, including five major public hospitals which account for a large but declining share of deliveries, due to more deliveries taking place in upgraded health centres and dispensaries in recent years [16]. Studies of facility-based maternal and perinatal mortality in 2007 reported high rates which appeared to have declined post-2015, possibly related to interventions such as Safe Motherhood Initiative at a national and subnational level in Tanzania [6, 16, 17]. Information on causes of child mortality in Dar es Salaam is limited to a few clinical studies [18, 19]. Coverage of maternal and new-born health interventions in Dar es Salaam has been higher compared to other areas [20, 21], and the living conditions are assumed to be better than in rural areas [22], but less information is available on how equitable the coverage of interventions and the living conditions are within Dar es Salaam across the different socio-economic status groups.

To improve the understanding of the extent to which extent and why the urban child mortality advantage has eroded, this study aimed to investigate levels and trends in child mortality over time comparing Dar es Salaam with urban/rural mainland and by socioeconomic status within Dar es Salaam. We also examined levels and trends of key determinants of child survival, including morbidity, nutritional status, health service coverage and living conditions within Dar es Salaam among the poorest and wealthiest and between Dar es Salaam and other urban and rural mainland populations in Tanzania using data from multiple sources [23]. To investigate into health system factors and particularly quality of care, we also assessed and compared availability and readiness of health facility services among all health facilities in Dar es Salaam and routine reports from three major regional referral hospitals within Dar es Salaam.

## Methods

### Study area

**Fig. 1:**
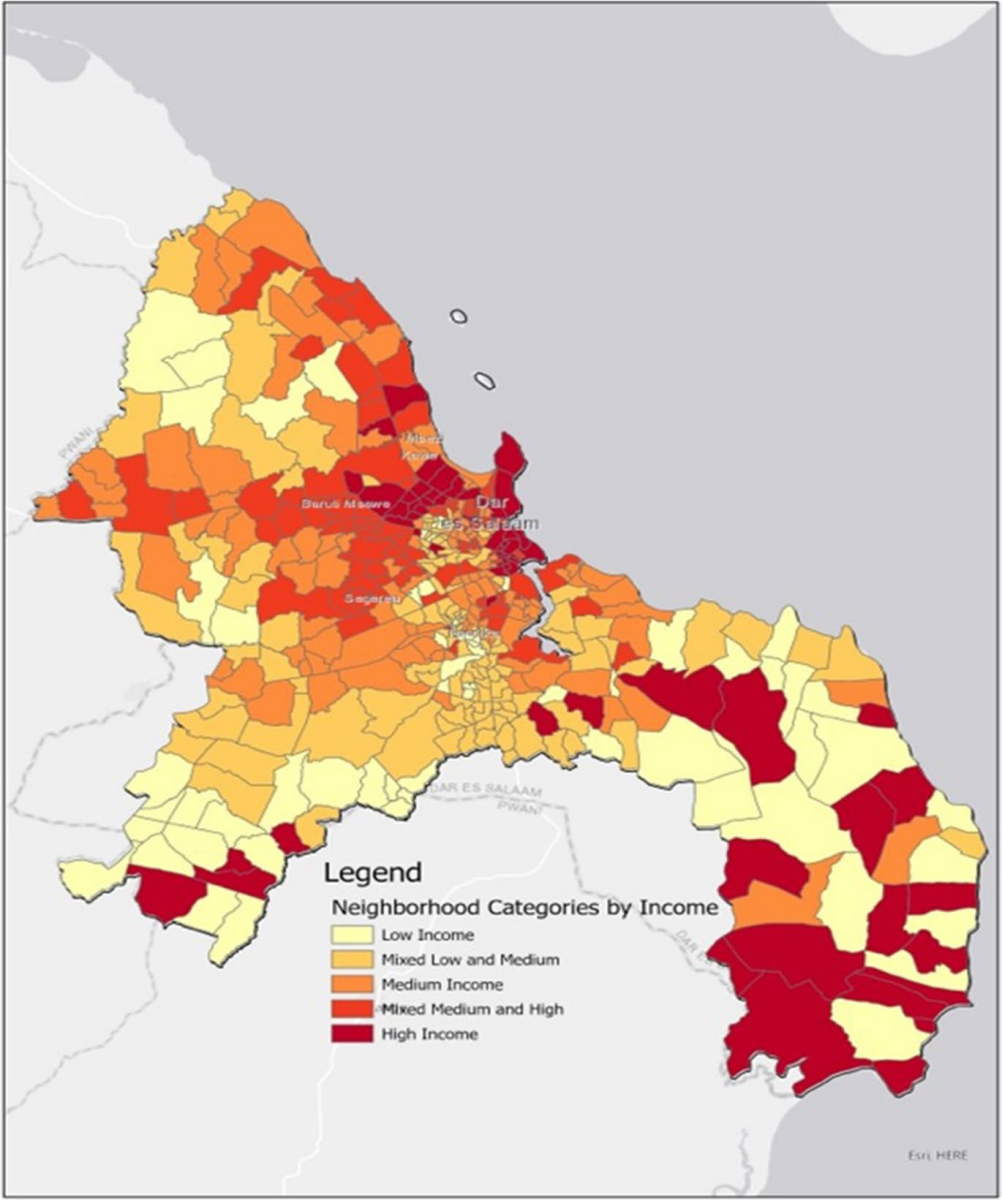
Map of Dar es Salaam region showing neighbourhood/streets categories by income levels Source: Centre for Sustainable, Healthy and Learning Cities and Neighborhoods, Tanzania 2020

Dar es Salaam city is subdivided into five municipalities: Kinondoni, Ubungo, Ilala, Temeke and Kigamboni, and 90 wards. The city occupies the whole Dar es Salaam region, one of 26 in Tanzania mainland, and there is no distinct rural zone [15]. The level of income varies within the city (Fig. 1) [24], over 60% of neighborhoods in Dar es Salaam were classified as low and medium income, with low-income neighborhoods located primarily in peripheral areas of the city from the Centre for Sustainable, Healthy and Learning Cities and Neighborhoods study.

### Data 

The main data sources included were national household surveys (Tanzania Demographic and Health surveys (TDHS) in 2004/05, 2010 and 2015/16), AIDS Indicator Surveys (AIS) and Malaria Indicator Surveys (MIS) to assess child mortality rates, and the coverage of key mother and child health indicators; health facility service assessments (Service Availability and Readiness Assessment (SARA) in 2017 and 2020) and routine health facility data (District Health Information System (DHIS2) for 2015–2021) were used for analysis of health system factors.

The TDHS, AIS and MIS employed a multi-stage sampling procedure, and its methods have been described in detail elsewhere [25]. The three TDHS surveys had weighted sample sizes of just over 10,000 women aged 15–49 years. In Dar es Salaam region, 412, 444 and 797 women aged 15–49 years, were interviewed in TDHS 2004/2005, 2010 and 2016, respectively. Birth history was used to collect data on mortality, and a health module gathers information on health and nutritional indicators of all children under 5 years of age. AIS and MIS were used to obtain information on HIV prevalence among women of reproductive age and malaria prevalence among children aged 6–59 months, respectively [26, 27].

The national SARA was conducted to assess and monitor health service availability, readiness and capacity in a two-stage sampling design. The first stage involved a random selection of one district from each region in the Tanzania mainland sample of facilities. In each selected district, all hospitals, health centres and a random sample of dispensaries were included in the assessment. Temeke and Kinondoni municipal councils were selected in Dar es Salaam region for SARA 2017 and 2019, with 93 and 111 health facilities (*N* = 549 and *N*=598 facilities in total), respectively. Further details on SARA methodology are explained elsewhere [28].

The Ministry of Health uses the District Health information system (DHIS2) to gathers monthly health facility reports from all health facilities on the Tanzania Mainland, including service delivery, morbidity and mortality [29].

### Conceptual framework

Child mortality was grouped into neonatal (0–28 days), post-neonatal (1–59 months) and under-5 mortality rate (0–59 months). The investigation into factors affecting child survival was based on the proximate determinants’ framework for child survival [23], as well as the WHO social determinants of health framework [30]. The outcome of interest, child mortality, is assumed to be primarily the result of deaths due to infections and malnutrition. The incidence and case fatality of these conditions are affected by the health and nutritional status of children and their mothers (such as maternal HIV, malaria, child stunting and wasting) [18, 31, 32], fertility, coverage of interventions (such as maternity care, immunization, treatment) [18, 31, 32] and living conditions (such housing, water and sanitation and household socioeconomic status) [22, 33].

Our analyses included the following indicators of vulnerability and exposure, as well as child-relevant treatment indicators:

- Health and nutritional status of children: stunting in children under 5 years, malaria prevalence in children 6–59 months based on a rapid diagnostic test
- Health of women of reproductive age: total fertility rate, HIV status
- Living conditions: improved water source, improved sanitary facility, housing conditions (with crowding defined as at least three persons sleeping in a single room)
- Coverage of interventions: antenatal care with contents (referred to as ANCq) [34], institutional birth coverage, C-section rate and a modified composite coverage index [35]

Indicator definitions are shown in the Appendix Table 5. To obtain a comprehensive picture, we calculated a modified composite coverage index (CCI) from the survey data as the mean of ten reproductive, maternal and child health indicators: four or more antenatal care visits (ANC4), skilled birth attendance, institutional birth coverage, Bacille Calmette-Guérin (BCG), pentavalent and measles vaccination coverage among children 12–23 months; care-seeking for fever and acute respiratory tract infections in children under 5 years; oral rehydration solution for treatment of diarrhoea in children under 5 years; insecticide treated net coverage among children under 5 and intermittent preventive therapy (IPT2) for malaria among pregnant women. Specific service readiness indices assessed in this study at a health facility level were antenatal care, basic emergency obstetric and new-born care (BEmONC), comprehensive emergency obstetrics and new-born care (CEmONC), immunization, child health prevention and curative services, essential medicine for children and malaria treatment.

### Analysis

All household survey-based analyses were stratified by areas of residence: Tanzania mainland (all urban and rural mainland), Dar es Salaam, other urban mainland (excluding Dar es Salaam) and rural mainland. Within Dar es Salaam, we disaggregated the analyses into the wealth tertiles (poorest 33%, middle and the richest 33%), using a household wealth index score. The index was calculated based on a city-specific asset score using principal component analysis. We opted for tertiles, rather than the more conventional quintiles, as the best way to deal with the challenges of the surveys' small sample size in the city, the general knowledge about the proportion of the population classified as poor in socioeconomic surveys, and the need to ensure a clear separation between the poor and the rich groups.

For health services, we used data from SARA surveys to compute the service readiness index for the indicators described above. The service readiness index was calculated as the mean from multiple domains of each indicator. The summary indices were presented as a percentage ranging from 0 to 100% (0-poor score, 100-good score). We reported the indices in the selected municipalities of Dar es Salaam and compared these to the overall Tanzania mainland results. For the routine health facility reports, we computed the average number of curative services (outpatient and admissions) per child per year and case fatality rates per 100 under-5 admissions. Also, we used the same indicators to compare Dar es Salaam with the mainland, using data from three regional referral hospitals serving the poorer population of Dar es Salaam. No statistical test was used in comparison of indicators from SARA surveys and health facility data as the comparison was only based on the observed numbers.

Child mortality was estimated using direct methods from women’s birth histories using data on date of birth of children, survival status and age at death of deceased children, for the 10-year period preceding the surveys. We specifically applied synthetic cohort method, where mortality probabilities are built up from probabilities calculated for specific age intervals [36]. Total fertility rates were calculated considering the number of births in the five years before the survey to women aged 15–49 years at the time of the birth. Standard errors for mortality and fertility were estimated through jackknife method, and 95% confidence intervals were obtained.

To assess differences in health status and coverage indicators across the stratifiers, we used chi-squared test (*X*^*2*^) of association or Fisher’s exact test (when expected cell count was < 5) to test if there was an independent association between the subgroups of interest. We compared Dar es Salaam city with other urban mainland, Dar es Salaam city with rural mainland and across the wealth tertiles. A *p*-value of *< 0.05* was considered statistically significant.

All analyses were conducted using Stata version 17.0 [37]. All estimates took into account the complex survey nature of the data, weighting for sampling probabilities and non-response, clustering and stratification done in the TDHS; Stata *svy* command was used accordingly.

### Ethical consideration

Ethical clearance to conduct studies under the Tanzania Countdown to 2030 collaboration was obtained from the National Institution for Medical Research in Tanzania with an ethical clearance number: NIMR/HQ/R.8a/Vol.IX/4099.

## Results

### Mortality

**Fig. 2:**
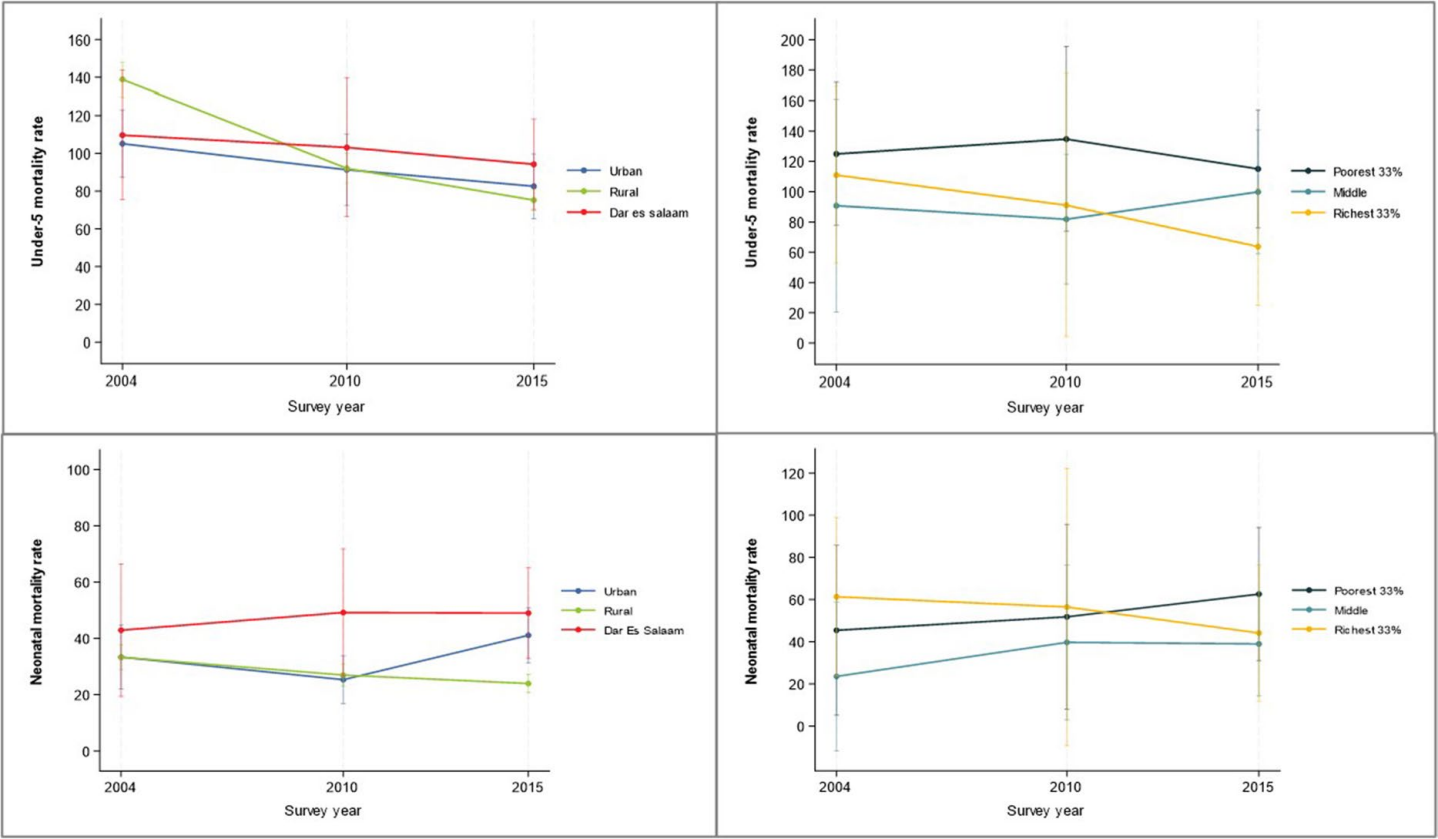
Under-five and neonatal mortality per 1000 live births by area of residence in Tanzania mainland (left panels) and wealth tertiles within Dar es Salaam (right panels) Source: TDHS 2004/05, 2010, 2015/16

The under-5 mortality rate declined by 41% in Tanzania mainland from 132 (95% CI: 124, 140) deaths per 1000 live births in 2004/05 TDHS to 78 (95% CI: 73, 84)) deaths per 1000 live births in 2015/16 TDHS (Appendix Table 6). The urban advantage in Dar es Salaam city and other urban mainland areas was substantial in TDHS 2004/05 with estimates of 110 (95% CI: 76, 144) and 105 (95% CI: 87, 123) deaths per 1000 live birth, respectively, compared to 139 in rural mainland (95% CI: 130,148). In the TDHS 2010, the differences among the three groups were small. The situation changed in the TDHS 2015/16, however, with  Dar es Salaam (94 deaths per 1000 live births; 95% CI: 70, 118) and other urban areas (83 deaths; 95% CI: 65, 100)) having higher mortality than rural mainland (75 deaths; 95% CI 70, 81), respectively (Fig. 2).

Differences in post neonatal mortality (1–59 months) were small in the two most recent surveys, but neonatal mortality was considerably higher in Dar es Salaam across the three survey rounds with a widening gap with rural mainland. In TDHS 2015/16, neonatal mortality rate was more than 50% higher in Dar es Salaam compared to rural areas, 49 (95% CI: 33, 66) vs 24 (95% CI: 21, 27) deaths per 1000 live births (Appendix Table 6).

Within Dar es Salaam, under-5 mortality was higher among the poorest compared to the richest tertile in all surveys, with also the middle tertile making little progress. None of the differences, however, were statistically significant. In TDHS 2015/16, compared to the richest tertile, the poorest tertile had a higher neonatal (63 vs 44 deaths per 1000 live births) and post neonatal mortality rate (61 vs 20 deaths per 1000 live births). In addition, the poorest-richest neonatal mortality rate gap (relative difference) in Dar es Salaam increased overtime from a mere 6% in TDHS 2004 to 29% in TDHS 2015/2016 (Appendix Table 6).

### Health and nutritional status of mother and child

Across all surveys, the prevalence of stunting, an indicator of chronic malnutrition, was significantly lower in Dar es Salaam compared to other urban and rural mainland areas (Table 1). In TDHS 2015/16, 12.7% of children under 5 years in Dar es Salaam were stunted compared to 26.5% in other urban mainland and 35.3% in rural mainland. Within Dar es Salaam, children from the poorest tertile households had higher stunting prevalence than those from the richest tertile households (18.3% and 5.8%, respectively), but still much lower than rural children.

**Table 1:** Mother and child indicators estimated from Tanzania Demographic and Health surveys, 2004/05, 2010 and 2015/16, HIV and malaria Indicators surveys, 2008, 2012, 2015 and 2017

Health and nutritional status of children								
	Child stunting (0–59 months)			Malaria prevalence (6–59 months)				
	2004/05 *N* = 7937	2010 *N* **=** 7480	2015/16 *N* **=** 9264	2012 *N* **=** 7717	2015 *N* **=** 8856	2017 *N* **=** 7177		
Area of residence								
Tanzania Mainland	33.4	37.6	31.8	9.9	15.6	9.5		
Dar es Salaam	12.2	16.5	12.7	3.6	1.0	3.6		
Other urban	26.6*	30.5*	26.5*	3.9	5.7*	3.5		
Rural	35.9^¥^	40.3^¥^	35.3^¥^	11.2^¥^	19.5^¥^	10.7^¥^		
Within Dar es Salaam								
Poorest 33%	16.6	20.6	18.3	5.0	1.1	5.4		
Middle	16.8	11.6	13.9	4.4	1.6	4.0		
Richest 33%	3.8	16.9	5.8	1.2	0.0	1.3		
*p value*	*0.096*	*0.444*	0.040	*0.395*	*0.532*	*0.422*		
Health of women of reproductive age								
	HIV status (women 15–49 years)			Total fertility rate (per 1000)				
	2004/05 *N* **=** 12,522	2008 *N* = 14,306	2012 *N* = 17,066	2004/05	2010	2015/16		
Area of residence								
Tanzania Mainland	5.8	5.9	5.3	5.7	5.5	5.2		
Dar es Salaam	7.3	9.4	7.0	2.6	3.0	3.2		
Other urban	8.9	9.5	8.1	4.2	4.0	4.0		
Rural	4.7^¥^	4.8^¥^	4.5^¥^	6.5	6.2	6.0		
Within Dar es Salaam								
Poorest 33%	7.9	13.8	7.8	2.8	3.4	4.1		
Middle	8.1	5.7	6.2	2.9	3.2	3.0		
Richest 33%	5.9	8.7	6.9	2.1	2.4	2.5		
*p value*	*0.511*	*0.126*	*0.733*					
Living conditions								
	Crowding (≥ 3 people in one room)		Unimproved water source			Unimproved sanitation		
	2010 *N* = 9375	2015/16 *N* = 12,246	2004/05 *N*= 9483	2010 *N* = 9375	2015/16 *N* = 12,247	2004/05 *N* = 9478	2010 *N* = 9373	2015/16 *N* = 12,247
Area of residence								
Tanzania Mainland	33.5	32.7	52.0	51.1	39.2	96.2	80.3	65.4
Dar es Salaam	32.3	32.3	20.1	18.9	16.8	90.8	59.0	6.2
Other urban	29.2	27.9	23.0	25.8	11.3	86.6	52.4	30.8*
Rural	34.7	34.4	63.2^¥^	60.9^¥^	52.1^¥^	99.2^¥^	89.6^¥^	86.4^¥^
Within Dar es Salaam								
Poorest 33%	40.4	40.5	24.6	24.4	24.2	98.0	78.8	14.7
Middle	34.8	43.0	19.9	16.6	13.1	93.9	70.9	2.6
Richest 33%	21.5	13.3	15.9	15.7	13.1	80.3	26.4	1.2
*p-value*	*0.028*	< *0.001*	*0.371*	*0.177*	*0.034*	*0.001*	< *0.001*	< *0.001*

*p-value comparing Dar es Salaam to other urban mainland (*p < 0.05); ^¥^*p*-value comparing Dar es Salaam to rural mainland areas (^¥^*p* < 0.05); *p-value*: shows comparison among wealth tertiles within Dar es Salaam

The prevalence of malaria among children 6–59 months in Dar es Salaam was lower than in other urban mainland areas and significantly lower than rural Tanzania. Within Dar es Salaam, malaria prevalence was higher among children in the poorest households, but even the prevalence in that tertile was lower than the malaria prevalence in rural Tanzania in the latest survey (5.4% vs 10.7%). HIV prevalence among women 15–49 in both Dar es Salaam and other urban mainland areas was higher than among rural women, across the years. Within Dar es Salaam, HIV prevalence was higher among the poorest in 2008 and 2012 surveys and the rate in that tertile was higher than the HIV prevalence in rural Tanzania. However, the differences across the tertiles in the city were not statistically different (*p*-values *> 0.05*). Total fertility was considerably lower in Dar es Salaam than in rural areas, while the poorest had a total fertility rate that was 1.6 children higher than the richest in the city.

### Household characteristics and living conditions

According to both the TDHS 2010 and TDHS 2015/16, about one-third (32.3%) of households in Dar es Salaam were overcrowded. Within the city, crowding increased with the level of poverty; households in the poorest tertile had almost twice the rate of crowding, compared to the richest one, and the gap increased over time (from 40% vs 21% in TDHS 2010 to 40% vs 13% in TDHS 2015/16) (Table 1).

The percentage of households with unsafe water or unimproved water supply was significantly lower in Dar es Salaam compared to rural mainland, but almost twice as common among the poorest households in the city compared to the richest ones.  According to the TDHS 2015/16, the proportion of households with unimproved sanitation declined substantially across the Tanzania mainland from the 2004/05 survey, and most prominently in Dar es Salaam to just 6.2% of households, ranging from 14.7% among the poorest to 1.2% among the richest (Table 1).

### Coverage of interventions

In all surveys, the maternal and child health coverage index was higher in Dar es Salaam (> 70%) than in other areas in Tanzania mainland. The coverage of the composite indicator of antenatal care with timeliness, intensity and contents of services (ANCq) was also substantially higher in Dar es Salaam. Both composite indicators showed that the poorest women were not at a disadvantage compared to other mainland areas.

The proportion of women delivering in health facilities was significantly higher in Dar es Salaam across all survey rounds compared other mainland areas (> 90%). The rate of cesarean section was significantly higher in Dar es Salaam and other urban areas compared to the overall mainland average and rural areas. Notably, the cesarean section rate among the poorest was less than 3% in the first two surveys but increased rapidly to 11.9% in TDHS 2015/16. Also, the use of cesarean section among the richest women became common (33.7%) in the same survey year (Table 2).

**Table 2:** Mother and child intervention coverage indicators estimated from Tanzania Demographic and Health Surveys, 2004/05, 2010 and 2015/16

Quality of maternal and new-born health care						
	ANCq (score ≥ 7)			C-section rate		
	2004/05 *N* = 5628	2010 *N* = 5405	2015/16 *N* = 6947	2004/05 *N* = 5628	2010 *N* = 5378	2015/16 *N* = 6908
Area of residence						
Tanzania Mainland	47.5	55.4	63.3	4.1	5.4	7.0
Dar es Salaam	91.5	95.8	92.3	7.1	11.9	17.7
Other urban	74.4*	82.4*	81.8*	8.6	9.4	10.5*
Rural	38.2^¥^	46.2^¥^	54.0^¥^	2.9^¥^	4.0^¥^	4.5^¥^
Within Dar es Salaam						
Poorest 33%	92.8	93.6	92.4	2.3	2.9	11.9
Middle	88.1	95.9	93.0	11.1	18.5	15.7
Richest 33%	95.6	100.0	90.9	8.0	19.3	33.7
*p*-*value*	*0.412*	*0.300*	*0.886*	*0.237*	*0.062*	*0.007*
Coverage of interventions						
	Composite coverage index (CCI)			Institutional deliveries		
	2004/05	2010	2015/16	2004/05 *N* = 5,628	2010 *N* = 5,378	2015/16 *N* = 6,908
Area of residence						
Tanzania Mainland	52.7	59.6	65.0	50.2	54.1	66.1
Dar es Salaam	72.5	70.7	77.7	91.9	89.5	94.9
Other urban	63.9	68.2	72.5	78.6*	83.4	85.6*
Rural	49.6	57.0	61.6	40.8^¥^	44.8^¥^	56.6^¥^
Within Dar es Salaam						
Poorest 33%	71.4	67.3	76.1	81.3	79.7	91.6
Middle	77.3	72.4	79.1	97.3	95.2	97.4
Richest 33%	68.5	74.9	76.2	100.0	100.0	98.1
*p*-*value*				*0.002*	*0.025*	*0.154*

*p-value comparing Dar es Salaam to other urban mainland (*p < 0.05); ^¥^*p*-value comparing Dar es Salaam to rural areas (^¥^*p* < 0.05); *p-value*: shows comparison among wealth tertiles within Dar es Salaam

### Facility assessments

The Dar es Salaam municipal councils (Temeke in 2017 and Kinondoni in 2020) had 1.0 and 1.1 health facilities per 10,000 population in the SARA 2017 and 2020, respectively, which was lower than the mainland as a whole (1.9 in 2017 and 1.5. in 2020) (Appendix Table 7).

The summary measures across several readiness indicators of the 2017 and 2020 SARA (Table 3) show little difference in terms of service readiness between mainland overall, urban mainland, rural mainland and Dar es Salaam. In 2020, for example, the general readiness score was 74% in Dar es Salaam and 66% in mainland Tanzania. Overall, Dar es Salaam facilities scored slightly higher for service readiness related to maternity services (ANC, BEmONC and CEmONC) and similar in child health services compared to mainland Tanzania. There was a difference of  -11 and  -5 for immunization readiness and malaria treatment readiness in 2020 with Dar es Salaam having lower scores compared to mainland Tanzania.

**Table 3:** Summary of readiness score of health facility assessments (%), SARA 2017 and SARA 2020, for overall mainland, urban and rural mainland, and Dar es Salaam region (Temeke in 2017, Kinondoni in 2020)

	2017					2020				
Indicator	Mainland	Urban	Rural	Dar es Salaam	Difference mainland-DSM	Mainland	Urban	Rural	Dar es Salaam	Difference mainland-DSM
Number of facilities						598	323	275		
General service readiness index	57	65	55	75	18	66	64	72	74	8
ANC readiness mean score	59	68	57	74	15	71	70	75	79	8
BEmONC readiness score	61	61	61	73	12	66	65	71	79	13
CEmoNC readiness score	68	75	64	74	6	51	42	69	69	18
Immunization readiness score	84	78	87	59	-25	86	91	76	75	-11
Child health prevention and curative	70	72	69	70	0	74	72	78	74	0
Essential medicines for children	57	61	56	64	7	55	55	57	54	-1
Malaria treatment readiness	67	65	68	60	-7	62	61	64	57	-5

### Health facility reports

According to DHIS2 facility reports from 2017 to 2021, the hospital admission rate per 100 children under 5 years was similar in Dar es Salaam (4.0% mean over the five years) to the mainland (4.5% mean). The case fatality rate per 100 admissions of children under 5 years could only be computed for the three main regional referral hospitals in Dar es Salaam as major underreporting was likely for all other facilities. In the three hospitals, 7.2% of all admissions died, three times higher than in the mainland, based on all reporting hospitals. Children in Dar es Salaam visited outpatient health services (for curative care) more frequently than overall for mainland: 1.2 and 0.7 visits per child under 5 per year, respectively. Stillbirth rates were 19.8 per 1000 births in all Dar es Salaam facilities, compared to 11.0 in mainland level, but were even higher (31.0) in the three regional referral hospitals in the city (Table 4).

**Table 4:** Under-5 health indicators for Dar es Salaam and mainland health facilities, DHIS2

	2017	2018	2019	2020	2021	Mean 2017–2021
Hospital admissions per 100 children under-5						
Mainland	4.4	4.4	5.1	4.8	3.8	4.5
Dar es Salaam	3.7	3.9	4.4	3.8	4.3	4.0
Case fatality per 100 admissions under-5						
Mainland	3.0	2.5	2.4	2.6	3.2	2.7
*Dar es Salaam (3 hospitals)	7.1	5.2	6.9	5.8	10.9	7.2
OPD visits per child under-5 per year						
Mainland	0.6	0.7	0.9	0.7	0.6	0.7
Dar es Salaam	0.8	1.1	1.5	1.3	1.5	1.2
Stillbirth rates (per 1000 births)						
Mainland	14.3	11.7	10.4	9.7	9.1	11.0
Dar es Salaam (all health facilities)	20.1	20.2	20.3	19.9	18.7	19.8
*Dar es Salaam (3 Hospitals)	25.2	26.9	27.6	33.2	41.8	31.0

*Three major public hospitals in Dar es Salaam: Ilala, Temeke and Amana

## Discussion

Tanzania experienced a large child mortality decline during the last two decades that was driven by a much more rapid decline in rural populations. Dar es Salaam made only piecemeal progress, and under-5 mortality was higher than in other urban areas and rural mainland Tanzania in the 2010 and 2015/16 surveys. The higher under-5 mortality is primarily due to a very high neonatal mortality in Dar es Salaam which has been the case since TDHS 2004. The mortality data suggest that Dar es Salaam children pay an urban penalty as living in the city may not confer the expected survival benefits associated with greater access to health and social services. Within the city, the poorest children, and to a lesser extent, those in the middle household wealth tertile, are bearing the brunt of the mortality penalty with higher levels of mortality compared to elsewhere in Tanzania mainland.

Our systematic assessment of health and living conditions provides few answers as to why this urban penalty has occurred. Overall, Dar es Salaam women have lower fertility rate, households have greater access to improved water and sanitation facilities however, similar levels of crowding with rural households. The gap between the poorest in Dar es Salaam and rural is small but still favours the city residents, except for crowding. City children, including the poorest, have far less stunting and lower malaria prevalence than rural children, even though differences are large within-city. HIV prevalence is higher in Dar es Salaam than rural mainland, but the differences are modest. Coverage of mother and child interventions in Dar es Salaam is higher than the mainland overall with little difference between the richest and poorest within the city. In TDHS 2015/16, no less than 90% of births in the city occurred in health facilities, which indicates that the explanation for elevated neonatal mortality could be found in the quality of health services.

The health facility assessments in 2017 and 2020 did not provide any further clues, however, as there were no major differences in service readiness between Dar es Salaam and elsewhere in Tanzania. We did observe that case fatality rates for under-5 child admissions were high in three regional referral hospitals in Dar es Salaam. Previous studies have documented high institutional maternal mortality and poor quality of care in large-volume and crowded facilities in Dar es Salaam [17, 38]. Although these three facilities play a major role in health service provision for the poorest in the city, it should also be noted that lower level and private hospitals also refer patients with severe illness to higher level hospitals such as Muhimbili National Hospital where the hospital records are not captured by the routine health facility data. Excluding the national hospital in this study is likely to under-estimate the case fatality rate among children under-5 in the city, and this deserves further inquiry.

Higher neonatal mortality in urban areas including Dar es Salaam is consistent with a few other studies conducted in sub-Saharan Africa, including Tanzania and Kenya that showed a significant reduction of the urban advantage over time when compared to rural areas [39–41]. This urban–rural difference in neonatal mortality may partly be attributed to the increased concentration of maternal and child health in rural areas, enabling them to catch up with urban areas. There is also a possibility of reporting bias due to greater underreporting of neonatal deaths in rural areas compared to urban areas. Quality of care may also explain the higher neonatal mortality rate in urban areas because even though there is a higher number of health facilities in them compared to rural areas, the quality of care may be lower due to crowding in health facilities [18, 19].

Research on causes of child mortality in Dar es Salaam is limited to clinical studies. A study comparing the diagnoses among sick children at outpatient visits showed similar morbidity patterns in Dar es Salaam and a rural site, with a wide array of bacterial, viral and parasitic pathogens [42]. Multiple studies in the national referral hospital in Dar es Salaam have shown a high rate of mortality among children with septicemia, partly associated with multi-drug resistance, both for neonates and older children [42–45]. A four-hospital study in 2017–2018 showed that malaria prevalence and in-hospital case-fatalities for children under-5 have declined while antimicrobial resistance and mortality due to bacteraemia increased [46]. A much better understanding of what is happening with neonatal and child care in health facilities is needed.

An important limitation of our analysis is the reliance on national household surveys which are designed to represent populations at regional and national level. Further disaggregation in the analysis such as the wealth groups within Dar es Salaam across indicators results in large sampling errors which makes the findings, especially on mortality, to be uncertain. Our analyses, however, gained strength from the consistency of results over the three surveys during 2004–2016. A second limitation concerns data availability which affected our choice of indicators. Several measures such as specific household living and environmental conditions, disease incidence, detailed health seeking behaviour and quality of care indicators were not available for this analysis. Injury-related mortality risks were not considered. We cannot dismiss an effect of differential underreporting of mortality, especially in the neonatal period, by rural residents. The high child mortality observed in Dar es Salaam compared to other Tanzania mainland areas and intra-city inequalities in indicators for health and living conditions need further understanding. We therefore recommend for further primary qualitative and quantitative studies be undertaken to examine these factors in more depth with adequate sample size that will allow more detailed data analysis.

## Conclusion

Children under 5 years of age in Dar es Salaam are affected by a substantive mortality penalty, erasing and even reversing a potential survival advantage since at least 2005. Notable progress occurred in rural mainland Tanzania that resulted in major mortality reductions, but this did not occur in the largest city of the country. The urban poor paid the highest price, as intra-city inequalities were large for almost all indicators. There is an urgent need for more investments into population-based and clinical research within Dar es Salaam as well as evidence-based program strategies that aim to reach the poorest in terms of disease prevention and high-quality health services.

## Appendix

Please see Tables 5, 6, and 7.

**Table 5:** Indicator definitions

Indicator	Definition	Denominator (Weighted number)
Stunting (< -2SD)	Children 0–59 months with weight for age below < -2SD	Children 0–59 months who were weighed
Malaria parasitemia	Children 6–59 months with positive blood slide or rapid diagnostic test	Children 6–59 months who were measured
HIV status	Proportion of women of reproductive age (15–49 years) tested HIV positive	All women living in households testing for HIV (AIDS Indicator Survey)
Crowding	Proportion of households with ≥ 3 people in one room	All households interviewed
Households with unsafe water source^1^	Proportion of households with unsafe water	All households interviewed
Households with unimproved sanitation facility^2^	Proportion of households with unimproved sanitation	All households interviewed
Composite coverage index	Mean coverage of coverage rate for the following reproductive, mother and child health indicators: 1) Antenatal care 4^th^ visit among live births in the last 5 years 2) Skilled birth attendance among live births in the last 5 years 3) Institutional deliveries among 4) Vaccinations (BCG, DPT3 and Measles) among children 12–23 months 5) Care-seeking for children with diarrhoea and acute respiratory tract infections in the last 2 weeks 6) Treatment of diarrhoea with ORS among children with diarrhoea in the last 2 weeks 7) Insecticide treated net coverage among children under-five (slept under ITN during the last night) 8) Two doses of Intermittent preventive therapy with Fansidar for pregnant mothers	
Quality Antenatal care (ANCq)	Proportion with quality ANC 1) At least one ANC visit 2) ANC visits with skilled provider 3) Total number of ANC visits 4) Blood pressure measured 5) Blood sample collected 6) Urine sample collected At least 2 shots of Tetanus Toxoid	Women with a live birth in the past 5 years prior the survey

^1^ Unsafe water sources-Unprotected dug well, unprotected spring, cart with small tank/drum, tanker truck, and surface water (river, dam, lake, pond, stream, canal, irrigation channels), bottled water

^2^ Unimproved sanitation facility -Public or shared latrine, Open pit latrine, Bucket latrine

**Table 6:** Under-five, post-neonatal and neonatal mortality per 1,000 live births by area of residence in Tanzania mainland and wealth tertiles within Dar es Salaam, TDHS 2004/05, 2010, 2015/16

Neonatal mortality rate (per 1000 livebirths)						
	TDHS 2004/05		TDHS 2010		TDHS 2015/16	
	Estimate	95% CI	Estimate	95% CI	Estimate	95% CI
Tanzania Mainland	34	30, 38	28	24, 31	29	26, 32
Dar es Salaam	43	19, 66	49	27, 72	49	33, 65
Other urban	33	22, 45	25	17, 34	41	31, 51
Rural	33	29, 38	27	23, 31	24	21, 27
Within Dar es Salaam						
Poorest 33%	45	5, 86	52	8, 96	63	31, 94
Middle	24	-12, 59	40	3, 76	39	14, 64
Richest 33%	61	24, 99	56	-9, 122	44	12, 76
Post-neonatal mortality rate (1–59 months) (per 1000 livebirths)						
	TDHS 2004/05		TDHS 2010		TDHS 2015/16	
	Estimate	95% CI	Estimate	95% CI	Estimate	95% CI
Tanzania Mainland	98	91, 106	65	58, 71	49	45, 53
Dar es Salaam	67	37, 96	54	26, 83	45	25, 65
Other urban	72	58, 86	66	48, 84	41	31, 52
Rural	105	97, 114	65	58, 72	51	46, 56
Within Dar es Salaam						
Poorest 33%	67	21, 113	42	12, 72	61	23, 99
Middle	80	36, 123	83	35, 132	52	28, 77
Richest 33%	50	-0.1, 99	35	-3, 72	20	0, 39
Under-five mortality rate (per 1000 livebirths)						
	TDHS 2004/05		TDHS 2010		TDHS 2015/16	
	Estimate	95% CI	Estimate	95% CI	Estimate	95% CI
Tanzania Mainland	132	124, 140	92	85, 100	78	73, 84
Dar es Salaam	110	76, 144	103	67, 140	94	70, 118
Other urban	105	87, 123	91	72, 110	83	65, 100
Rural	139	130, 148	92	84, 100	75	70, 81
Within Dar es Salaam						
Poorest 33%	125	78, 172	135	74, 196	115	76, 154
Middle	91	20, 161	82	39, 125	100	59, 141
Richest 33%	111	53, 169	91	4, 178	64	25, 102

**Table 7:** Summary of SARA 2017 and 2020 showing health facility densities comparing mainland and Dar es Salaam

	SARA 2017		SARA 2020	
	National sample	Dar es Salaam (Kinondoni District)	National sample	Dar es Salaam (Temeke District)
Number of facilities sampled	549	111	598	93
Density	1.87 per 10,000	1.01 per 10,000	1.5 per 10,000	1.1 per 10,000
